# Matrix Mechanics as Regulatory Factors and Therapeutic Targets in Hepatic Fibrosis

**DOI:** 10.7150/ijbs.37500

**Published:** 2019-09-07

**Authors:** Guobao Chen, Bin Xia, Qiang Fu, Xiang Huang, Fuping Wang, Zhongmin Chen, Yonggang Lv

**Affiliations:** 1 School of Pharmacy and Bioengineering, Chongqing University of Technology, Chongqing 400054, P. R. China.; 2 Chongqing Technology and Business University, Chongqing 400067, P. R. China; 3 Key Laboratory of Biorheological Science and Technology (Chongqing University), Ministry of Education, Bioengineering College, Chongqing University, Chongqing 400044, P. R. China; 4 Mechanobiology and Regenerative Medicine Laboratory, Bioengineering College, Chongqing University, Chongqing 400044, P. R. China

**Keywords:** liver fibrosis, matrix stiffness, targeting, mechanotransduction, myofibroblasts, hepatic stellate cells

## Abstract

The hallmark of liver fibrosis is excessive extracellular matrix (ECM) synthesis and deposition that improve liver matrix remodeling and stiffening. Increased matrix stiffness is not only a pathological consequence of liver fibrosis in traditional view, but also recognized as a key driver in pathological progression of hepatic fibrosis. Cells can perceive changes in the mechanical characteristics of hepatic matrix and respond by means of mechanical signal transduction pathways to regulate cell behavior. In this review, the authors first classify causes of liver matrix stiffening during fibrotic progression, such as higher degree of collagen cross-linking. The latest advances of the research on the matrix mechanics in regulating activation of HSCs or fibroblasts under two-dimensional (2D) and three-dimensional (3D) microenvironment is also classified and summarized. The mechanical signaling pathways involved in the process of hepatic matrix stiffening, such as YAP-TAZ signaling pathway, are further summarized. Finally, some potential therapeutic concepts and strategies based on matrix mechanics will be detailed. Collectively, these findings reinforce the importance of matrix mechanics in hepatic fibrosis, and underscore the value of clarifying its modulation in hopes of advancing the development of novel therapeutic targets and strategies for hepatic fibrosis.

## 1. Introduction

Liver fibrosis is a major cause of morbidity and mortality worldwide. Liver fibrosis occurs after a person experiences injury or inflammation in the liver. Many of the causes can lead to fibrosis, including autoimmune hepatitis, biliary obstruction, iron overload, nonalcoholic fatty liver disease, viral hepatitis B and C, and alcoholic liver disease 1. Among them, liver fibrosis in Western societies is mainly caused by nonalcoholic fatty liver disease (NAFLD), while in China it is mainly caused by viral hepatitis. After liver fibrosis occurs, the cells, components, blood vessels, and microstructures in the liver will change in different degrees. Histologically, fibrosis is marked by disruption of the hepatic architecture and deposition of abnormal extracellular matrix (ECM). Among them, matrix stiffening is not only the pathological result of liver fibrosis, but also one of the important cues for promoting the progression of liver fibrosis. On the one hand, matrix stiffness, as an important feature and marker of pathological results of liver fibrosis, has received more and more attention and recognition in the clinical diagnosis of liver fibrosis. On the other hand, matrix mechanics has also been shown to promote the progression of liver fibrosis. Recently, studies have been proposed to target matrix mechanics as a new target for fibrosis treatment to explore new therapeutic approaches in liver fibrosis.

**Figure 1:**
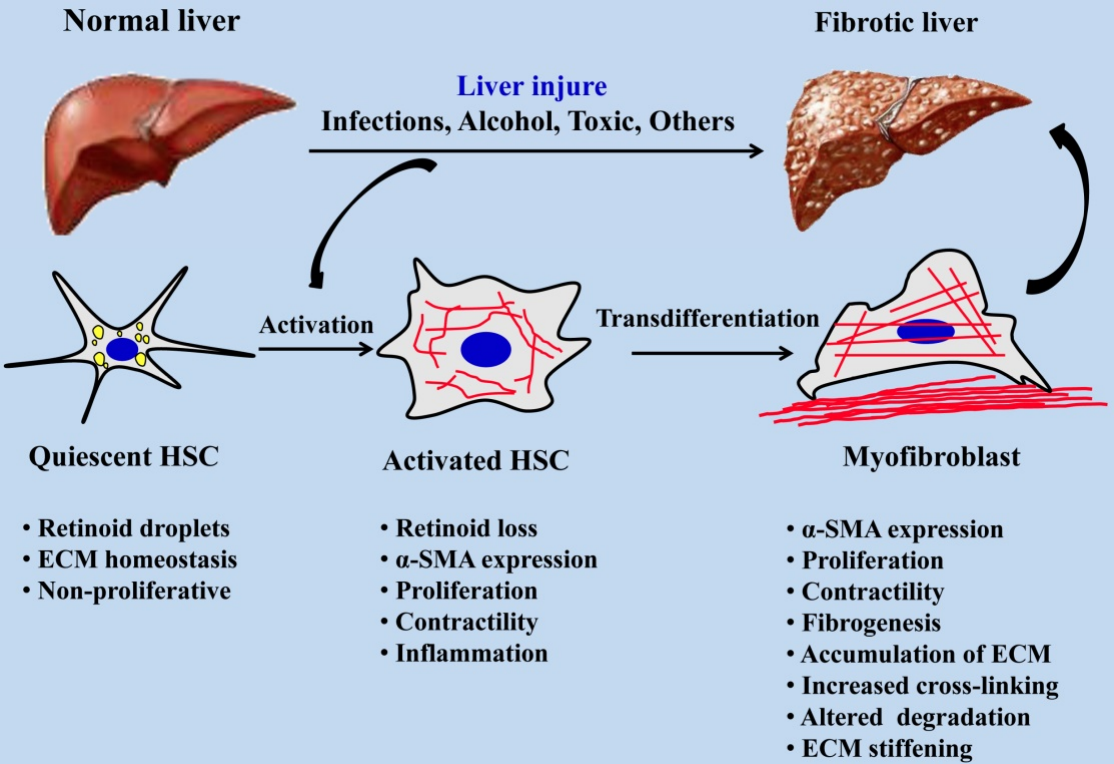
Activation process of quiescent HSCs into myofibroblasts during hepatic fibrosis.

Myofibroblasts are the main effector cells that produce ECM in the process of fibrosis, and hepatic stellate cells (HSCs) are one of the main cell sources of myofibroblasts in the liver, accounting for about 82~96% 2. The activation of HSCs is an important event in the process of hepatic fibrosis. After liver injury, quiescent HSCs are activated and differentiate into myofibroblasts. After excessive proliferation of myofibroblasts, a large amount of ECM such as collagen is synthesized, accompanied by increased matrix cross-linking and insufficient ECM degradation, eventually leading to liver fibrosis 3. HSCs are non-parenchymal cells in the liver and are rich in lipid drops when they are in a quiescent state. As shown in Figure 1, once the liver is damaged, HSCs are activated and lose fat droplets, and their proliferative and contractile abilities increase and release cytokines such as inflammatory and fibrosis promotion 4. HSCs will migrate after continuous activation, and a large amount of metalloproteinase tissue inhibitor-1 (TIMP-1) produced by autocrine and paracrine can effectively reduce the activity of Matrix metalloproteinase-2 (MMP-2) by binding to matrix MMP-2, thereby inhibiting the degradation of ECM by MMP-2, resulting in abnormal excessive deposition of ECM 5. With the increasing pathological degree of hepatic fibrosis, the stiffness of hepatic tissue is increasing, the hepatic fibrosis in different pathological periods corresponds to different hepatic tissue stiffness 6, 7, and the stiffness of liver has become one of the important indexes in the clinical diagnosis of pathological staging of hepatic fibrosis.

The pathological stage of hepatic fibrosis is generally divided into 5 grades: F0 is non-hepatic fibrosis, F1 is mild hepatic fibrosis, F2 is moderate hepatic fibrosis, F3 is severe hepatic fibrosis, and F4 is cirrhosis. Corpechot et al. 8 examined the liver stiffness of 168 patients by transient elastography and found that the stiffness thresholds for different stages of liver fibrosis were 6.5±1.0 kPa (F1), 8.1±0.7 kPa (F2), 10.8±2.3 kPa (F3), and 13.7±1.7 kPa (F4), respectively. Georges and colleagues 9 examined liver stiffness at different stages of rat liver fibrosis and found that the shear modulus of the liver after fibrosis ranged from 3 kPa to 22 kPa, significantly higher than the shear modulus of normal liver. These findings all indicate that liver stiffness is significantly positively correlated with the degree of fibrosis.

Here we start with introducing various cues acting on liver matrix stiffening during the pathological progression, mainly including the changes of ECM, cell mechanics, and inflammation. We will describe the latest advances of the research on the matrix mechanics in regulating liver fibrosis under two-dimensional (2D) and three-dimensional (3D) microenvironment. Because accumulated evidences show that several signaling pathways are involved in the process of the liver matrix stiffening, such as the YAP/TAZ signaling pathway, which is proved to play an important role during liver fibrosis. Finally, some potential therapeutic concepts and strategies based on matrix mechanics are described.

## 2. Causes of Liver Matrix Stiffening during Fibrotic Progression

**Figure 2:**
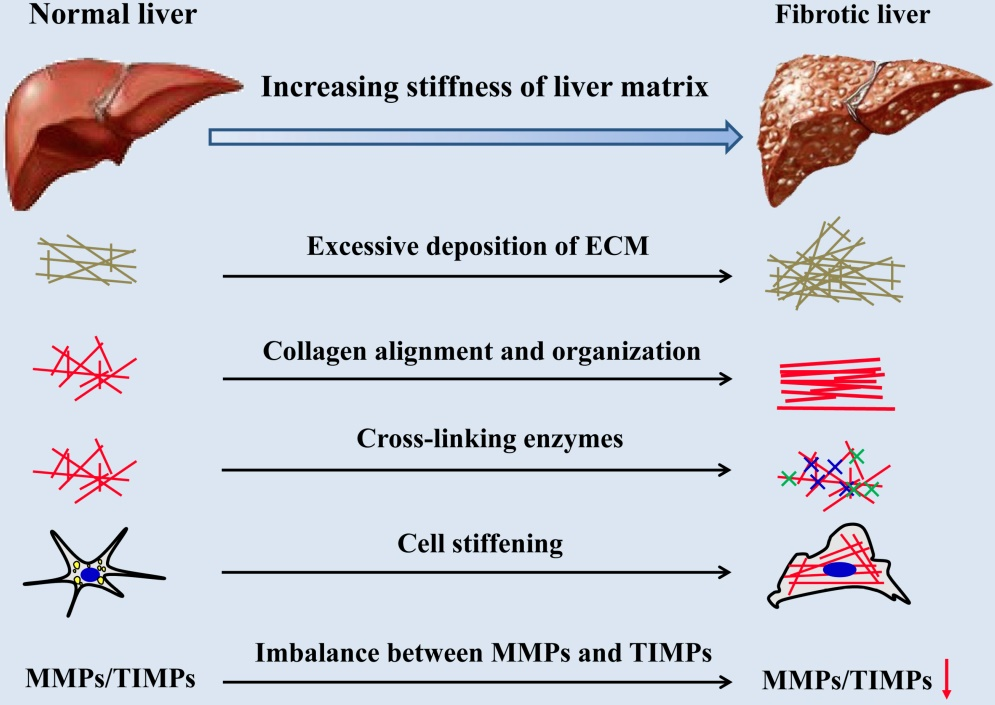
Causes of liver matrix stiffening during fibrotic progression.

There are many cues for the increase of hepatic matrix stiffness during the progression of fibrosis (Figure 2), including excessive deposition, organization and cross-linking of ECM, imbalance of MMPs and its inhibitors expression, increased stiffness of intrahepatic cells and hepatic inflammatory microenvironment. Stiffening of the liver matrix caused by these factors will further affect the fibrotic behavior of intrahepatic cells and form positive feedback.

### 2.1 Excessive deposition of ECM, mostly collagen type I

Many factors have been shown to lead to change in the stiffness of the liver. Among them, the major reason of elevated matrix stiffness during liver fibrosis is dysregulated matrix synthesis and remodeling. Normally, the most important structural ECM components in liver matrix are collagen, proteoglycans, laminin, fibronectin, and matricellular proteins 10, and the ECM is degraded and reformed in a balanced way to maintain tissue homeostasis. In advanced stage of hepatic fibrosis, the liver contains approximately 6 times more ECM than normal, including collagen type I, III, and IV, fibronectin, undulin, elastin, laminin, hyaluronan, and proteoglycans 11. For instance, the low-density basement membrane-like matrix of the space of Disse in normal liver is composed mainly of collagens IV and VI. After liver injury, disruption of this matrix and replacement by fibrillar collagens occur; this matrix is composed of collagen type I and III and fibronectin 1, 12. In a carbon tetrachloride (CCl_4_) induced rat liver fibrosis model, quantitative analysis of Sirius red staining revealed total collagen deposition in the liver increased as fibrosis progressed, which is 4.94% of the total tissue in liver sections at 4 weeks, 8.25% at 6 weeks, and 9.11% at 8 weeks 13. Desai and colleagues 14 did the measurements orthogonal to fibrotic tracts and found that liver matrix was significantly stiffer in regions approaching fibrillar collagen deposition and returned to near normal rigidity in areas remote from it within the same lobule. Hepatic fibrosis is the results in excessive production of ECM, mostly collagen type I 15. It has been proved that the content of collagen type I plays a key role in changing the mechanical characteristics of tissue and ECM, and the stiffness of various tissues and organs is positively correlated with the content of collagen type I 16, 17. Therefore, the massive accumulation of collagen type I is an important contributing factor in the local stiffening of the ECM in progressive liver fibrosis.

### 2.2 Collagen alignment and organization

In addition to change the amount of collagen deposited in the liver matrix, the alignment of the collagen fibrils is also contributing significantly to the alteration of the stiffness of liver matrix. Matrix stiffness can affect the contractility of growing cells and the cellular contractility further increase the contractility and alignment of the collagen fibrils in the liver matrix 18, 19. Furthermore, the collagen matrix alignment *in vivo* can significantly improve the strength and stiffness of the entire matrix relative a matrix that is a disordered matrix 20. The collagen matrix in the aligned region has greater stiffness due to its better structural order 21 and many *in vitro* studies prove that mechanical strength of ordered or aligned collagen nanofibers is significantly higher than that of the disordered nanofibers 22, 23. For example, the electrospun collagen type I with the principal axis fibril alignment indicated an average load of 1.17 ± 0.34 N at failure with a peak stress of 1.5 ± 0.2 MPa. The average modulus for the longitudinal samples was 52.3 ± 5.2 MPa. In cross fiber orientation, the peak load at failure was 0.75 ± 0.04 N with a peak stress of 0.7 ± 0.1 MPa. The modulus across the fiber long axis was 26.1 ± 4.0 MPa 23. Another study also found significant differences in elastic modulus between the aligned and random collagen type I scaffolds, with the aligned groups (567 ± 134 kPa) having significantly greater elastic modulus than random groups (349 ± 85.5 kPa) 24. Fibrosis is associated with alterations of interstitial flow because of vessel hyperpermeability and/or angiogenesis, Ng and colleagues 25 demonstrated that low levels of interstitial flow (0.1-0.3 dyn/cm^2^) can induce collagen fibers to align perpendicular to the shear force direction and promote fibroblast transdifferentiation into matrix-producing myofibroblasts* via* triggering the expression of transforming growth factor-β1 (TGF-β1).

Besides the variation of orientation of collagen fibrils during the pathological progression of hepatic fibrosis, the change of collagen organization also found to be involved in the reversal of liver fibrosis, thus affecting liver matrix mechanics. Several researchers proposed that the large collagen fibers in liver matrix can break into smaller collagen fragments during the fibrosis regresses, and this change may further affect the local mechanical properties of liver matrix and make mechanical effects on the cells in liver matrix 26. A new study, using atomic force microscopy (AFM) to compare the characteristics of normal and idiopathic pulmonary fibrosis lung tissue, found that the diameter of collagen fibers in the pulmonary matrix after fibrosis was significantly smaller, the swelling rate decreased, and the stiffness of the matrix increased significantly 27. These findings suggest that the abnormal changes of collagen fibrils in liver matrix during fibrosis can remarkably regulate the mechanical properties of the tissue, and the underlying mechanism that lead to these abnormalities are crucial for researchers to understand of the fibrotic mechanisms and to provide new therapeutic targets.

### 2.3 Collagen cross-linking enzymes and matrix mechanics

In addition to the promotion of matrix stiffness by the increase in collagen content, the degree of cross-linking also affects the mechanical properties of the matrix. The collagen cross-linking enzymes appear to act a pivotal role in fibrosis or cancer progression by regulating the mechanical properties of the matrix 28. In liver fibrosis, several studies have demonstrated that lysyl oxidase (LOX) may represent the major cross-linking activity for collagen 9, 29. LOX has also been shown to interact with fibronectin, which in turn increases the catalytic activity of LOX, thereby increasing the cross-linking of collagens, and subsequently, matrix stiffness 30. The cross-linking endows the resistance to proteolytic degradation and further promotes excess deposition of ECM. Increased liver stiffness early after injury is associated with increases in LOX-mediated collagen cross-linking. Liu and colleagues 31 found that LOX had the functional contribution to stabilize collagen in liver fibrosis progression or reversal. In this work, the proportion of insoluble collagens increased from 5.7% in healthy tissue to 14.7% and 19.1% in fibrotic tissue of C57Bl/6J mice treated with CCl_4_ for 3 and 6 weeks, while the soluble collagens decreased from 92% in healthy controls to 84% and 79% in fibrotic tissue at 3 and 6 weeks. The treatment of LOX inhibitor β-aminopropionitrile (BAPN) decreases collagen stability during liver fibrosis progression and facilitates fibrosis reversal after CCl_4_-induced advanced liver fibrosis 31. In another insightful work, BAPN-treated fibrotic mice approved the LOX inhibitor can effectively disrupt the collagen fibril networks and significantly reduce the stiffness of collagen fibril 32. Besides the animal models, elevated expression and activity of LOX family members are also observed in sera of patients with hepatic diseases, for instance, liver fibrosis and cirrhosis 33.

Transglutaminases (TGases) are another type collagen cross-linking enzymes in progressive liver fibrosis 34, 35. Some *in vitro* analyses verified that collagen type I gel cross-linked by the reactions of transglutaminase can effectively elevate the stiffness of gel compared to the uncrosslinked group 36, 37. This treatment can not only improve the mechanical characteristics and stability of the tissue matrix, but also give the matrix resistance to proteolytic degradation. In an earlier study 34, the increase in transglutaminase detected by immunohistochemistry in fibrotic, compared to normal, liver is in agreement with the increase in transglutaminase expression during experimental liver fibrogenesis 38, 39. Recently, Tatsukawa and colleagues 40 found that the TGase 1 activity may be involved in the functional modification of intracellular proteins, whereas TGase 2 activity contributes to the stabilization of extracellular proteins during liver fibrosis.

### 2.4 MMPs and their specific inhibitors in liver matrix mechanics

MMPs are a family of over 24 zinc-dependent endopeptidases capable of degrading virtually any component of the ECM, especially collagen that is responsible for matrix structure and mechanical properties in the ECM. The degradation has demonstrated that is a very critical process during the tissue or organ development, repair, fibrosis, and so on 41. In normal livers, MMPs and their natural inhibitors, TIMPs, are always in a dynamic balance to maintain the normal expression and deposition levels of the ECM in the liver. In general, TIMPs are a family of at least four identified physiological inhibitors (TIMP-1, TIMP-2, TIMP-3, and TIMP-4) capable of regulating proteolytic activities of MMPs in tissues 42. However, after fibrosis occurs, this balance will be broken and the profibrogenic effects of TIMP-1 are thought to be mediated *via* preventing collagen degradation through inhibition of MMPs 43. The imbalance between the MMPs and TIMPs will affect the degradation and deposition of collagen, and these variations in ECM composition likewise have a profound influence on the mechanical characteristics of the liver matrix 44. By detecting and comparing the liver stiffness and the expression of TIMPs in 29 patients with acute liver failure, the expression of hepatic stiffness and TIMP-1 in patients was found to be significantly higher than that of healthy controls 45. Meanwhile, the compensatory increase in MMP expression is overcome by a parallel decrease in the MMP/TIMP ratio both during the onset and 7 days after the patients first presented with acute liver failure 45. And in another study, Latronico and colleagues 46 measured the circulating levels of different MMPs and TIMPs in HCV, it was found that TIMP-1 levels were significantly higher in HCV subjects compared to healthy donors and were correlated with liver stiffness. The findings show that the expression of MMP and TIMPs will change in the process of hepatic fibrosis, thus affecting the content change of collagen and other ECM components in liver matrix, eventually altering the matrix stiffness of liver and affecting the process of hepatic fibrosis.

### 2.5 Cell stiffening contributes to the matrix mechanical properties of liver

Just as different tissues have their specific elastic modulus in the body, different cells also have unique stiffness in the tissue or organ. It has been found in previous studies that diseased cells are stiffer than healthy cells in certain pathologies 47-49. For instance, the study found that the elevated aortic stiffness with aging is not only due to changes in the ECM, but also because of the increased stiffness of vascular smooth muscle cells in the old male monkeys 47. Similarly, in a recent study on non-idiopathic pulmonary fibrosis, the fibroblasts derived from patients with this disease were found to have higher stiffness than their normal counterparts 48. And in an *in vitro* model for liver cirrhosis, the stiffness of human hepatoma-derived HepG2 cells was significant increased after treatment with three known induction factors (collagen substrates, alcohol, and CCl_4_) 49. Moreover, changes in the stiffness of hepatocytes can further affect the hepatic functions, such as glucose secretion 49. In these studies, the increase in cell stiffness was mainly due to changes in the expression of the cytoskeletal proteins, for example, the increase in stiffness of vascular smooth muscle cells and lung fibroblasts was mainly due to the increased expression of α-smooth muscle actin (α-SMA). Another study also supports this conclusion from the side, that is, the normal lung can maintain 81% of the original stiffness of the liver after decellularization, while the lung with idiopathic pulmonary fibrosis retains only 44% of the original lung stiffness after decellularized treatment 50. This finding shows that the cells in the fibrosis lungs contribute more to the stiffness of the whole lung than the cells in the normal lungs. During the fibrosis process, quiescent HSCs are activated to become myofibroblasts and expressed more α-SMA, so there is reason to believe that the stiffness of myofibroblasts may be higher than that of quiescent HSCs.

### 2.6 Hepatic inflammation microenvironment and liver stiffness

Although fibrosis is certainly a major contributing factor to liver stiffness, other characteristics of liver diseases, like inflammation may potentially affect liver stiffness. A recent study of pediatric patients with a variety of liver diseases similarly found that the correlation between alanine aminotransferase and transient elastography result was stronger among those with inflammatory diagnoses and F0/F1 fibrosis 51. Inflammation is one of the most typical features of chronic liver disease of viral, alcoholic, fatty and autoimmune origin. It occurs at all stages of liver disease, including fibrosis, sclerosis and the development of liver cancer 52. Previous studies have contributed to promote the understanding of the connection between liver inflammation and fibrosis 53, 54. In a paper analyzing liver inflammation and liver stiffness by comparing 325 clinical samples, it was found that inflammation had a significant effect on liver stiffness values in patients with mild fibrosis, but not in patients with significant fibrosis 54. Moreover, it was also found that patients with the same fibrosis stage but higher inflammation grade tended to have higher liver stiffness measurement values 54. After the occurrence of hepatic fibrosis is often accompanied by intrahepatic inflammation, inflammation not only affects the function of the liver and immune microenvironment, inflammation caused by the secretion of intrahepatic ECM has also been shown to be involved in the changes in the stiffness of the intrahepatic matrix.

## 3. Matrix Mechanics and Progression of Liver Fibrosis

Given the important role that matrix mechanics plays in the progression and reversal of liver fibrosis, more and more researchers are beginning to focus on how to construct matrix microenvironment *in vitro* that can simulate occurrence and development of liver fibrosis* in vivo*, including 2D and 3D conditions. In the liver, the most studied cells in response to changes in matrix stiffness are HSCs and portal fibroblasts. These two types of cells are the major cellular sources of collagens and LOX in normal liver and early liver injury 55. They can sensitively perceive and respond to alteration in the stiffness of the matrix in which they are located. Previous studies suggest that matrix stiffness affects multiple biological behaviors and properties of cells and tissues, including cell adhesion, proliferation, migration, differentiation, and so on 56, 57. Therefore, analysis of the influences of matrix stiffness on the progression of liver fibrosis will provide the researchers with new insights in understanding pathological mechanisms and potential therapeutic targets for hepatic fibrosis.

### 3.1 2D matrix stiffness

**Figure 3:**
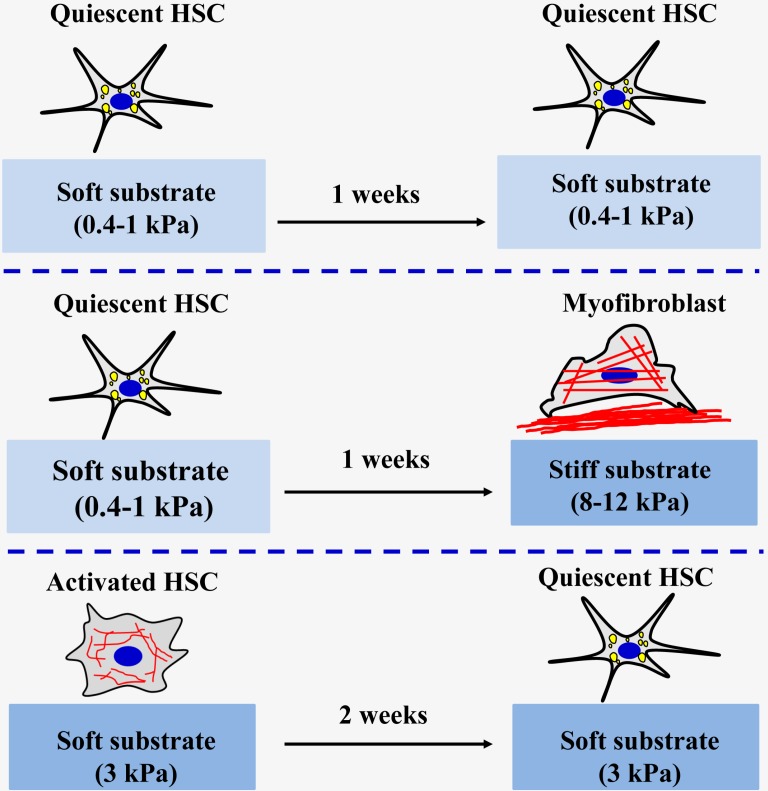
The effect of matrix stiffness on the activation of HSCs.

At present, most existing studies on engineering the matrix mechanical microenvironment of intrahepatic cells have been focused on 2D substrates. Polyacrylamide (PA) gel is the most classic and commonly used polymer to create elastic cell culture substrates with tunable stiffness by adjusting the acrylamide monomer and crosslinker content in PA gels 58, 59. The research team led by Wells has found that a stiffer matrix microenvironment can induce portal fibroblasts 60 and HSCs 61 to differentiate into myofibroblasts (Figure 3). Portal fibroblasts were cultured on PA gels support of variable stiffness (400 Pa~12 kPa) coated with thin layers of collagen type I to observe the myofibroblastic differentiation in different substrates 60. It was found that the softest substrate (400 Pa) can maintain the phenotypic appearance of freshly isolated fibroblasts. Conversely, the substrate with a stiffness of 12 kPa can promote the myofibroblastic differentiation of portal fibroblasts by elevating the expression of α-SMA at gene and protein levels. Moreover, the cell area also increases as the matrix stiffness increases. It was also found that TGF-β and matrix stiffness play a synergistic role in promoting the mRNA expression of procollagens I and III 60. Similar results were also found in HSCs cultured on PA gels of variable stiffness, ranging from 0.4 to 12 kPa 61. Activated HSCs are thought to play a central role in secreting large amounts of ECM and regulating the degradation of matrix. The results showed that HSCs seeded on the softer substrates (0.4-1.0 kPa) for one week could maintain their quiescent state, including rounded morphology and lipid droplets. On the stiffer substrates (8-12 kPa), the lipid droplets of the HSCs disappeared, the morphology changed from circular to spreading, and α-SMA expression level and organization of stress fibers increased, indicating that the HSCs had activated into myofibroblasts 61. Quiescent HSCs initially planted on the softer substrates were then covered with the glass coverslips for 3 days could increase cell spreading area and loss of lipid droplets. This finding indicated that HSCs on softer substrates were viable and the stiffness sensing was dynamic. The study also specifically pointed out that the substrate stiffness is more decisive than the chemical properties of substrate when determining HSCs phenotypes *in vitro* 61. Exogenous TGF-β addition is not sufficient to promote an increase in the level of required expression of HSCs on the softer substrates (0.4 kPa). However, high-matrix stiffness was needed for the expression of α-SMA and collagen type I, no matter of TGF-β exposure, although addition of TGF-β significantly enhanced the effects of stiffness on the expression of α-SMA 61. Therefore, the above findings under 2D conditions proved that change of liver matrix mechanics drives the pathological progression of fibrosis. Additionally, the studies also found that 2D matrix stiffness can effectively regulate the proliferation of hepatocellular carcinoma cells and chemotherapy tolerance. Increasing matrix stiffness (12 kPa) promotes proliferation and resistances to chemotherapy, whereas a soft environment (1 kPa) induces reversible cellular dormancy and stem cell characteristics in hepatocellular carcinoma 62.

In addition to the classic PA hydrogels for the preparation of different stiffness substrates, the research team led by Wells and Burdick using methacrylates modified hyaluronic acid macromolecules and sequence of addition of dithiothreitol and then crosslinking by UV light induced radical polymerization to construct different stiffness substrates to mimic normal and fibrotic livers and investigate the effects and mechanism of soft or stiff substrates on the activation of HSCs 63-65. It was found that the high stiffness hydrogel substrate (24 kPa) can significantly increase the spreading area of rat HSCs, loss of lipid droplets, expression of high levels of α-SMA and collagen type I, and the low stiffness hydrogel substrate (2 kPa) can maintain lipid droplets of HSCs and expresses high levels of peroxisome proliferator-activated receptors γ (PPARγ) 63. Additionally, considering that the mechanical microenvironment of the cells in the fibrosis process is dynamic, this research group also innovatively studied the effects and mechanisms of dynamic matrix stiffening 64 or softening 65 on the progression and reversal of liver fibrosis. The stiffening from 1.75 kPa to 33.0 kPa of matrix resulted in myofibroblast activation of HSCs, which is a common feature of pathological fibrosis. They also revealed that the timing of substrate stiffening strongly influenced cell fate and delayed stiffening further promoted myofibroblast activation 64. Similarly, when the liver matrix becomes soft, it may also play an important role in the reversal of liver fibrosis. HSCs were cultured on tissue culture plastic obtained a myofibroblast phenotype and then maintained the activated phenotype onto the stiff hydrogel substrate (20 kPa) 65. Compared to the cells on the stiff control (20 kPa), when the activated cells are cultured on stiff-to-soft (20 kPa to 3 kPa) hydrogel substrate, the area of the cells is reduced and the expression levels of a-SMA and Yes-associated protein/Transcriptional coactivator with PDZ-binding motif (YAP/TAZ) are also decreased at 2 weeks. This result indicates that when the environment in which the activated cells grow from stiff to soft, the activated cells are reversed to a certain extent, but this reversal is not complete. Together, these findings demonstrated that matrix mechanics have great effects on fibrosis regression and reversal and that simulating the dynamically changing mechanical signals can help to better mimic the real environment of cells* in vivo*.

Besides the matrix stiffness sensing by HSCs in pathologic liver matrix stiffening, HSCs can also sense and response the matrix proteins in their culture system. Saums et al. 66 showed a new cell culture system combining layer-by-layer (LbL)-assembled films of different native matrix proteins with mechanically tunable substrates and to investigate the role of a role for both the matrix environment and substrate stiffness in myofibroblastic differentiation of HSC.

### 3.2 3D matrix stiffness

Cells are exposed to a solid microenvironment that fully surrounds them in 3D environments, while cells in 2D platform are typically exposed to a solid, flat surface on the basal side and to liquid at the apical surface. To recapitulate intrahepatic cells behaviors and responses in both physiological and pathological conditions of liver, engineering 3D spatiotemporal matrix mechanical microenvironments *in vitro* may help investigators to explore and discover the relationship between the matrix mechanical characteristics of the liver and its fibrosis occurrence and reversal.

To investigate the relationship between 3D matrix stiffness and proliferation, survival of hepatic cells, Bomo and colleagues 67 fabricated the 3D collagen matrices with different stiffness to explore the responses of human hepatocarcinoma cells (Huh7) to them. Collagen type I scaffolds with two different stiffness were formed by controlling the concentrations of collagen solution. It was found that the concentrations of 0.75 mg/mL and 1.5 mg/mL were used to form the stiffness of 1 kPa (soft scaffold) and 3.5 kPa (stiff scaffold), respectively. The increase from 1 kPa to 3.5 kPa can be a good simulation of the elevation of liver matrix stiffness during the liver fibrosis development* in vivo* 67. After 3 days of culture, Huh7 cells on the 3D stiff matrix were non-spread and exhibited spherical clusters. On the contrary, cells appeared a good spreading growth and developed monolayer-like structures on the soft collage matrix. Cells' spreading under 3D matrix stiffness is exactly opposite to the stiffness of the 2D matrix, which is directly related to the different ways in which cells are in contact with the matrix in under these two dimensions. Moreover, the survival and proliferation of Huh7 cells on different stiffness in 3D collage matrix were also evaluated in this work. It is found that the proliferation of Huh7 cells and normal hepatocytes on stiff collage matrix is higher than that in the soft collage matrix and 2D control by the colorimetric MTT assay. Furthermore, the stiff matrix can increase the biotransformation activities of Huh7 and Human hepatocytes in 3D as compared to 2D cultures and 3D soft matrix 67. This study points out the different effects of matrix stiffness on liver cells under 2D and 3D microenvironments, but the difference between the collagen scaffold and the composition of the liver matrix under physiological or pathological conditions is still large, and the 3D scaffold systems which can better simulate the microenvironment of hepatic matrix mechanical microenvironments need to be further developed. Ma and colleagues 68 used the liver decellularized ECM and a rapid digital light processing (DLP)-based 3D printing method to construct a new 3D liver matrix with tailored mechanical features to mimic the pathological mechanical properties of hepatocellular carcinoma. Mechanical characteristics of these 3D scaffolds can be modulated by controlling the light exposure time. Particularly, three different exposure times of 10 s, 20 s, and 40 s were selected to fabricate 3D scaffolds with stiffness values of 0.5 kPa, 5 kPa, and 15 kPa, which each corresponds to the softer than healthy liver range (soft), healthy liver range (medium), and cirrhotic range (stiff), respectively. The influence of scaffold composition can be eliminated by adjusting the light exposure time to change the stiffness of the scaffold. Compared with low stiffness and medium stiffness, the study found that HepG2 cells were encapsulated in high-stiffness scaffolds for 3 and 7 days to limit cell viability and growth. In addition, the stiffness of the scaffold has also been found to regulate cell migration and invasion by regulating the expression of cellular MMP2 and MMP9, that is, HepG2 in the high-stiffness scaffold endocrine more MMP2 and MMP9, thus further promoting the potential migration and invasion ability of HepG2 68. This nature-derived ECM scaffolds with regionally matrix mechanical features can provide the natural composition, biomimetic structure and more physiologically relevant tissue mechanical properties to serve as a better platform for liver disease research. In addition to directly acting on intrahepatic cells to affect liver fibrosis, matrix stiffness has also been found to promote HSCs activation by regulating angiogenesis, thus affecting the process of hepatic fibrosis. Recently, a research group led by Du 69 has constructed a 3D microfibrotic niche to mimic fibrotic liver matrix and utilized them to investigate how matrix mechanics affect the formation of capillary-like structures and its influence on the progression of liver fibrosis. This 3D microfibrotic niche consisted of liver sinusoid endothelial cells (LSECs), cultured on an underlying 2D polyethylene glycol (PEG) hydrogel substrate with varying stiffness, and an overlaid 3D collagen type I hydrogel, which was embedded with HSCs. It was found that the softer PEG hydrogel substrates with stiffness in the range 140-610 Pa showed typical pro-angiogenic properties as seen in early-stage fibrosis, whereas the stiffer ones (>1.2 kPa) exhibited random migration, sparsely distributed focal adhesion complexes and leaky vessels as observed during late-stage fibrosis 69, 70. Importantly, the stiffness of early-stage 3D microfibrotic niche was significantly increased by condensing of collagen fibrils by angiogenesis, and resulted in activation of HSCs by LSECs. Liu and colleagues 69 found the activation of HSCs by the mechanical force *via* interactions with collage fibrils and a cell membrane-bound discoidin domain receptor 2 (DDR2) receptor by using *in vitro* and *in vivo* models. The innovative fibrotic microniches developed and the findings from this excellent research work highlight the dynamic properties of fibrosis progression and the contribution of angiogenesis to fibrosis in the environment of different matrix mechanical signals.

## 4. Matrix Mechanics and Mechanotransduction Pathways in Liver Fibrosis

In this pathological process of gradual stiffening of the hepatic matrix, the cells in the liver matrix perceive changes in the biophysical characteristics of their microenvironment and activate the mechanotransduction pathways, thus converting the external mechanical stimuli into biochemical signals, ultimately guiding cells behaviors. How matrix mechanics influences and regulates cell fate from the macroscopic to the microscopic scales is still a black box that is not fully open, but some studies have found that some proteins involved in the mechanotransduction pathways play a role in this process.

As a classical signaling pathway, the TGF-Smad signaling pathway has been shown to be an important signaling pathway involved in the progression of fibrosis in matrix stiffness regulation. Myofibroblasts contraction can release the latent TGF-β1 from latent stores in local stiff matrix and still remains latent in the comparably soft matrix. Because the deformation caused by cell contraction on a soft substrate is absorbed and protected the large potential complex against conformational changes, and on a stiff substrate, this deformation is transmitted by integrin to induce the release of TGF-β1 after deformation of the large potential complex 71, 72. The released TGF-β1 feeds back by binding to its receptor and promotes development of fibrosis. Because activated TGF-β1 is a potent fibrogenic cytokine that promotes myofibroblastic differentiation, this finding suggests that stiff matrix may regulate myofibroblastic differentiation through an extrinsic mechanotransduction pathway in which stress fiber-generated contractile forces in response to matrix stiffening are transduced across the cell membrane and converted into the fibrogenic signal by activation of latent TGF-β1, resulting in myofibroblastic differentiation 73.

The activity of Rho-associated protein kinase (ROCK) of fibrosis fibroblasts has been shown to be increased by matrix stiffening 74, 75, and the contraction of myofibroblasts is dependent on Rho/ROCK/myosin light chain pathway activation 76 RhoA expression of lung fibroblasts at the mRNA level and protein level was higher on the stiff matrix (20.80±2.52 kPa) by the elevated stiff matrix than that in the soft matrix (0.52±0.09 kPa) 74. Consistent with results of RhoA, a stiff matrix also promotes the expression of α-SMA at gene and protein level and induces myofibroblast differentiation. Recently, Dou et al. 77 also demonstrated that substrate stiffness can activated AKT signal pathway by RhoA to induce phosphorylation of p300 and translocate to the nucleus of HSCs and affect the tumor metastasis by increasing expression of paracrine factors such as CXCL12. In this process, the protein expression of RhoA of human HSCs was increased by the matrix stiffness from 0.4 kPa to 25.6 kPa and promoted the nuclear accumulation of p300 and HSCs activation, suggested that RhoA is required of stiffness-induced phosphorylation of p300 77.

Recently, the YAP-TAZ signaling pathway is proved to be another important signaling pathway to participate in the progress of matrix stiffness regulation of hepatic fibrosis 64, 78-80. In liver fibrosis, YAP is activated in HSCs in response to matrix stiffening 64-65, 80. In mouse fibrotic livers, the stiffened matrix causes the integrin clustering and then activates the kinase activity of focal adhesion kinase (FAK). The activated FAK signal stimulates the ROCK-dependent actin remodeling and the formation of stress fibers. Finally, ROCK-myosin-II mediates the contraction of activated HSCs or fibroblasts and then drives the translocation of YAP-TAZ from the cytoplasm into the nucleus 81. The activation of YAP-TAZ in the nucleus results in inducing the expression of profibrotic genes and increasing the expression of α-SMA and excessive matrix deposition, which are associated with the generation of myofibroblasts 78. Immunohistochemical examination of fibrotic areas of livers from patients with hepatitis C 80 and lung tissue from patients with idiopathic pulmonary fibrosis (IPF) 82 found that the myofibroblasts in the fibrotic region with strong nuclear staining of YAP and TAZ. By analyzing the local stiffness of continuous slices of fibrosis tissue and the immunostaining of TAZ, the researchers also found that the part of TAZ positive nucleus and the areas of elevated tissue stiffness were co-localized. The findings demonstrated that increased stiffness of fibrotic tissue* in vivo* can promote YAP-TAZ nuclear accumulation and transcription activity in activated HSCs or myofibroblasts, promote collagen and fibronectin deposition and cause ECM stiffening, and eventually form a positive feedback loop with negative consequences 82.

## 5. Targeting the Matrix Mechanics in Liver Fibrosis

Matrix stiffening is a defining characteristic of hepatic fibrosis. While traditionally viewed as an endpoint, matrix stiffening is now recognized to develop early during fibrosis initiation, and to contribute prominently to disease progression through mechano-activation of myofibroblasts derived from fibroblasts or HSCs. Based on the importance of matrix mechanics in the current process of liver fibrosis, studies have been conducted to investigate whether targeting liver matrix mechanics has the potential to treat or even reverse liver fibrosis.

### 5.1 Reduce or reverse the stiffness of the liver matrix

Obviously, the most direct way to target the stiffness of the hepatic matrix is to inhibit and slow the increase of liver stiffness in the process of hepatic fibrosis or even reduce the stiffness of liver until it returns to normal liver stiffness. From this point of view, by influencing or reducing the factors that can promote the stiffening of the liver matrix, the purpose of reducing the stiffness of the liver matrix can be achieved, thereby inhibiting cell activation, alleviating or reversing liver fibrosis. Firstly, the purpose of reducing the stiffness of the liver is achieved by targeting the degree of cross-linking of the liver matrix. The LOX family of enzymes plays important roles in collagen cross-linking, contributing to both the increase in tissue stiffness and degradation resistance of collagen-rich matrices. Previous preclinical models reveal a striking reduction in tissue stiffness and fibrosis with use of the nonspecific LOX inhibitor BAPN 83. In addition to direct targeted LOX studies, inhibition of lysyl oxidase-like-2 (LOXL2) raised during hepatic fibrosis has also been shown to be effective in relieving hepatic fibrosis and promoting fibrosis reversal 84, 85. LOXL2 was shown to mediate the crosslinking of collagen and the fibrotic matrix stabilisation during the hepatic fibrosis process 85. The degree of cross-linking and stability of collagen directly modulates the stiffness of the entire liver matrix, thereby affecting the activation or inactivation of hepatic progenitor cell (HPC). It was direct assessed using a stepwise collagen extraction assay and found that the anti-LOXL2 antibody AB0023 treatment significantly suppressed the increase in crosslinked collagen, with a 45% reduction in insoluble fraction between 6 and 12 weeks of thioacetamide (TAA) administration and a 20.8% reduction of overall crosslinked collagen, compared with the control 85. LOXL1, another member of the LOX family, is thought to have the function of promoting the crosslinking of elastic proteins 86. Recently, a new study has confirmed that suppressing the expression of LOXL1 through silencing techniques can prevent the progression of cirrhosis by reducing the crosslinking of elastin in the hepatic matrix 87.

In addition, collagen, as one of the main components in the fibrotic matrix, plays a pivotal role in regulating the mechanical characteristics of the hepatic matrix. Therefore, targeting to reduce the synthesis of collagen 88 in the liver matrix during fibrosis or to degrade the excessive deposition of collagen by collagenase 89 has the potential to reduce the stiffness of hepatic matrix and achieve the goal of slowing down the process of hepatic fibrosis.

### 5.2 Reduce or inhibit the response of cells to stiffening of the liver matrix

Cells can sense and respond to the stiffness of their surrounding matrix 90. In liver, HSCs or fibroblasts can be activated by the elevated stiffness of matrix, in turn, upregulates ECM deposition and stiffness, constituting a positive-feedback loop 91. Therefore, it may be a potential way to ameliorate fibrosis by interfering with, reducing or inhibiting the responses of mechanosensitive cells in the liver to the stiffening of the matrix 92. The small Rho GTPases provide a functional linkage between mechanosensing through integrin-based adhesion and the actomyosin cytoskeleton by generating traction forces. ROCK is the major downstream effector of Rho that drives cell contractility and is a mediator of fibrotic pathologies. The ROCK activity of fibroblasts can be activated by increasing the stiffness of lung matrix during fibrosis, inducing the differentiation of fibroblasts into myofibroblasts, increasing the expression of α-SMA, and enhancing the actin cytoskeletal polymerization 74, 93. ROCK-mediated effects during fibrosis are mechanosensitive. Therefore, the inhibition of ROCK by means of drugs or inhibitors can alleviate the fibrosis process caused by the elevated matrix stiffness, which disrupts or blocks the responses of cells to tissue stiffening. Several studies have confirmed the inhibition of ROCK signaling by its inhibitors (such as Y-27632 and fasudil) attenuates stiffness-induced α-SMA expression and fiber assembly in myofibroblasts 74, 93-94.

After the initiation of fibrosis, YAP/TAZ will be activated by matrix stiffness and further promote the transformation of fibroblasts or HSCs into myofibroblasts, increase the deposition of collagen by the positive feedback loop that elevates the stiffness of matrix microenvironment and leads to YAP activation 80. Knockdown of YAP levels by transfecting siRNAs or pharmacological inhibition of YAP by verteporfin can effectively inhibit the activation of HSCs *in vitro* and can prevent the fibrogenesis in a CCl_4_-induced liver fibrosis in mice 80. Omega-3 polyunsaturated fatty acids (ω-3 PUFAs), such as docosahexaenoic acid (DHA) and eicosapentaenoic acid (EPA), demonstrate promising efficacy in alleviating hepatic fibrosis by targeting YAP to reduce the expression of pro-fibrogenic genes in activated HSCs and fibrotic liver 95. The findings demonstrated that YAP activation undertakes a central role of YAP/TAZ in the fibrogenic cascade, and the selection of a suitable method to target nuclear transcription of YAP/TAZ in cells during fibrosis may also be a potential anti-fibrotic pathway.

Enhanced contractility of myofibroblasts is an important phenotypic feature of myofibroblast differentiation during fibrosis. Meanwhile, quiescent tissue fibroblasts become activated to a contractile, myofibroblast matrix secreting phenotype and ultimately increase the tissue stiffness 96. For instance, Torr and colleagues 97 found that collagen matrix formation by myofibroblasts is dependent on the assembly of a fibronectin matrix and the assembly of fibronectin fibril by myofibroblasts requires contractile genes expression. Contractile genes expression is a necessary condition for the development of a contractile phenotype in myofibroblasts. Among them, the α-SMA gene is a key contraction gene that is raised in myofibroblasts and the expression of α-SMA may be the key to promote the accelerated assembly of fibronectin matrix by myofibroblasts 97. Therefore, disruption of the α-SMA by siRNA knockdown affects the contraction of myofibroblasts, thus reducing the assembly of fibronectin matrix into fibrillary ECM. In addition, a recent study in airway fibrosis has also found that by simultaneously utilizing relaxin and LOX inhibitors to target myofibroblastic contractility and matrix stiffness, respectively, showed that the deposition of collagen could be significantly reduced and the re-epithelialization of remodeled airways could be promoted 98.

## 6. Conclusion and Future Outlook

Although previous studies confirm that several mechanically sensitive proteins are involved in the progression of liver fibrosis, these key proteins are commonly expressed in many tissues and organs of the body, participating in other normal physiological activities of the human body, inhibiting or blocking them may cause other diseases or dysfunction in the body. Therefore, the development of higher specificity and more accurate targeted drugs or small molecular inhibitors is still a key and difficult point in this field. As an important parameter for the progress of liver fibrosis, non-invasive and accurate detection of local stiffness of the liver matrix *in vitro* is one of the important prerequisites for the development of diagnosis and treatment targeting liver matrix mechanics, especially in the initial stage when the hepatic matrix begins to stiffen. Direct intervention or reduction of the stiffness of the liver matrix by appropriate physical or chemical methods, reducing or blocking the promotion of liver fibrosis by matrix mechanics may avoid the off-target effect of the use of drugs to some extent. Considering the heterogeneity of liver matrix mechanics, future studies also need to construct a more accurate culture platform* in vitro* that matches the mechanical heterogeneity of the liver matrix to facilitate the study of liver fibrosis and provide more accurate pharmaceutical screening platform.
